# Low-Dose Rapamycin Treatment Increases the Ability of Human Regulatory T Cells to Inhibit Transplant Arteriosclerosis *In Vivo*

**DOI:** 10.1111/j.1600-6143.2012.04065.x

**Published:** 2012-08

**Authors:** J Hester, A Schiopu, S N Nadig, K J Wood

**Affiliations:** Nuffield Department of Surgical Sciences, Transplantation Research Immunology Group, University of OxfordUK

**Keywords:** Cell therapy, rejection, humanized mouse model, tolerance, T_reg_

## Abstract

Regulatory T cells (T_reg_) are currently being tested in clinical trials as a potential therapy in cell and solid organ transplantation. The immunosuppressive drug rapamycin has been shown to preferentially promote T_reg_ expansion. Here, we hypothesized that adjunctive rapamycin therapy might potentiate the ability of *ex vivo* expanded human T_reg_ to inhibit vascular allograft rejection in a humanized mouse model of arterial transplantation. We studied the influence of combined treatment with low-dose rapamycin and subtherapeutic T_reg_ numbers on the development of transplant arteriosclerosis (TA) in human arterial grafts transplanted into immunodeficient BALB/c*Rag2*^−/−^*Il2rg*^−/−^ mice reconstituted with allogeneic human peripheral blood mononuclear cell. In addition, we assessed the effects of the treatment on the proliferation and apoptosis of naïve/effector T cells. The combined therapy efficiently suppressed T-cell proliferation *in vivo* and *in vitro*. Neointima formation in the human arterial allografts was potently inhibited compared with each treatment alone. Interestingly, CD4^+^ but not CD8^+^ T lymphocytes were sensitive to T_reg_ and rapamycin-induced apoptosis *in vitro*. Our data support the concept that rapamycin can be used as an adjunctive therapy to improve efficacy of T_reg_-based immunosuppressive protocols in clinical practice. By inhibiting TA, T_reg_ and rapamycin may prevent chronic transplant dysfunction and improve long-term allograft survival

## Introduction

The mammalian target of rapamycin (mTOR), a serine/threonine protein kinase, is inhibited by the immunosuppressive drug rapamycin. mTOR plays a key role in the regulation of cell proliferation, adhesion and survival by integrating information from the cell's environment 1–3). By targeting mTOR, rapamycin inhibits the proliferation of many cell types including T cells, one of the key cellular mediators of rejection following transplantation. T cells are a heterogenous population of lymphocytes with different subsets having different functional capabilities. Moreover, T cells exhibit plasticity enabling some populations to change their functional properties depending on the environmental cues they receive both as they differentiate, as well as when they function *in vivo* (4). Each T-cell subset demonstrates a differential sensitivity to mTOR inhibition (5). Thus, the impact of rapamycin therapy *in vivo* may be different depending on the composition of the T-cell compartment in the host, the microenvironment in which a T cell is functioning, and the duration and dose of rapamycin therapy.

Regulatory T cells (T_reg_) play important roles in immune homeostasis and in the induction and maintenance of tolerance to self antigens, thereby preventing autoimmunity. Treg may also contribute to the induction and maintenance of tolerance to foreign antigens, including donor alloantigens in the context of transplantation (6). Both naturally occurring and alloantigen induced CD4^+^FOXP3^+^ T_reg_ have been shown to be able to control rejection and graft-versus-host disease and there is evidence that they participate in the development of specific unresponsiveness to alloantigens *in vivo* in mice and in humans (7). T_reg_ are thus being developed as a potential cellular therapy in cell and solid organ transplantation (8).

To provide proof of concept data to support the translation of T_reg_ therapy to the clinic, we have previously used a humanized mouse model to investigate the functional capabilities of naturally occurring T_reg_ to prevent allograft rejection *in vivo*. We have shown that *ex vivo* expanded human CD4^+^FOXP3^+^ T cells can prevent the development of transplant arteriosclerosis (TA) in transplanted human vessel allografts and the rejection of human skin allografts (9,10). Moreover, using the humanized mouse model of arterial transplantation, we demonstrated that the enrichment of human CD4^+^FOXP3^+^ T cells expressing low levels of CD127 (alpha chain of the IL-7 receptor) and high levels of CD25 (alpha chain of the IL-2 receptor) produces a population with increased regulatory activity after expansion *ex vivo* compared with enrichment protocols based on CD25 expression alone (10).

In contrast to the immunosuppressive, anti-proliferative effects that rapamycin treatment has on naïve/effector T cells, the drug promotes expansion of T_reg_ when cultured in the presence of high concentrations of IL-2 *ex vivo* (11). It has recently been reported that in the resting, anergic state T_reg_ are characterized by increased activity of the mTOR pathway, induced by the cytokine-like proinflammatory hormone leptin (12). Transient inhibition of mTOR with rapamycin followed by T-cell receptor (TCR) stimulation rendered T_reg_ highly susceptible to proliferation even in the absence of exogenous IL-2 (12). These results are in line with data demonstrating that after rapamycin-induced depletion, T_reg_ recover the ability to proliferate in response to antigen faster than conventional effector T cells, leading to an increase in the T_reg_:effector T-cell ratio (13).

Here we hypothesized that rapamycin could be used as adjunctive therapy *in vivo* to enhance the ability of human T_reg_ to prevent transplant rejection when only suboptimal doses of T_reg_ are available. We have investigated the influence of combined therapy using low-dose rapamycin and subtherapeutic numbers of *ex vivo* expanded human CD127^lo^CD25^+^CD4^+^FOXP3^+^ T_reg_ on allograft rejection, as demonstrated by TA development in the humanized mouse model of arterial transplantation. We show that T_reg_ and rapamycin can act together to suppress CD4^+^ and CD8^+^ T-cell proliferation *in vivo* and *in vitro*. Addition of rapamycin to T_reg_ therapy *in vivo* inhibits interferon gamma (IFNG) production and potently reduces neointima formation in transplanted human vessel allografts compared with each treatment alone.

## Methods

### Mice

Immunodeficient BALB/c *Rag2*^−/−^*Il2rg*^−/−^ (H2^d^) mice, lacking T, B and NK cells were purchased from Charles River Laboratories and housed under specific pathogen-free conditions. The mice were included in the experiments between the ages of 6 and 10 weeks. The animals were bred and maintained in the Biomedical Services Unit at the John Radcliffe Hospital (Oxford, UK) and were treated in strict accordance with the Home Office Animals (Scientific Procedures) Act of 1986.

### Arterial transplantation and tissue analysis

Transplantation of human arterial segments in BALB/c *Rag2*^−/−^*Il2rg*^−/−^ mice reconstituted with human peripheral blood mononuclear cells (PBMC) was performed as previously described (10). In brief, side branches of the internal mammary artery (IMA) were collected during cardiac bypass surgery under the ethical reference number 04/Q1605/89. Informed consent was obtained from all subjects. The arterial grafts were transplanted into the abdominal aorta of recipient mice by an end-to-end anastomosis technique. The day after transplantation, recipients were reconstituted intraperitoneally (i.p.) with 10 × 10^6^ Ficoll–Paque-purified PBMC from healthy blood donors, alone or in combination with 1 × 10^6^ donor-matched CD127^lo^T_reg_ expanded *ex vivo*. HLA analysis was performed for all vessel and PBMC donors to ensure allogenicity. Some of the recipients were additionally treated with 300 μg/kg rapamycin injected i.p. on day 7, 8 and 10 after transplantation.

The arterial grafts were recovered under anesthesia 30 days after transplantation snap-frozen in OCT (Sakura Finetek, the Netherlands) and cryostat sectioned at a 10-μm thickness. After drying, sections were fixed in 100% acetone for 10 min at 4 °C and stored in −80 °C until further use. For morphometric analysis, the sections were stained with Miller's Elastin/van Gieson and the percentage of the lumen occupied by the neointima, termed intimal expansion, was calculated using the following formula:% Intimal expansion = (AI/AI + AL) × 100, where AI is the area of the neointima and AL is the luminal area.

The degree of human lymphocyte reconstitution of the transplanted mice was verified at the time of recovery by flow cytometry analysis of single-cell splenocyte suspensions using antibodies against human CD3 (eBioscience, San Diego, CA, USA), CD4 (Caltag; Invitrogen, Carlsbad, CA, USA), CD8, CD25, CD45 and CD127 (BD Bioscience, Oxford, UK). Only mice with >1% engraftment of human CD45+ cells reported to total splenocyte count were considered to be fully reconstituted and were included in the study.

### Assessment of in vivo cellular proliferation in response to alloantigen

BALB/c *Rag2*^−/−^*Il2rg*^−/−^ mice were reconstituted with 10 × 10^6^ human PBMC i.p. and received human skin allografts collected during plastic surgery as previously described (9). Skin was obtained with ethical approval from the Oxfordshire Research Ethics Committee (study number 07/H0605/130) and with full patient consent. The mice were then allowed to reconstitute for 14 days. On day 14 we injected i.v. 10 × 10^6^ donor-matched CFSE (carboxyfluorescein diacetate succinimidyl ester)-labeled human PBMC, alone or in combination with 1 × 10^6^ unlabeled CD127^lo^Treg expanded *ex vivo*. In addition, some of the mice were treated with 300 μg/kg rapamycin injected i.p. on days 14, 15 and 17. On day 19, spleens were harvested and the intensity of CFSE staining in human CD4^+^ and CD8^+^ T lymphocytes was assessed by flow cytometry. The undivided T lymphocytes were quantified as percentage CFSE^hi^ cells of the total CFSE positive population.

### Isolation and expansion of human T_reg_ cells

Human T_reg_ cells were sorted and expanded as described previously (10). In brief, human PBMC have been isolated from buffy coats obtained from healthy volunteers using Ficoll–Paque (GE Healthcare, Bio-Sciences, Uppsala, Sweden) gradient centrifugation. CD4^+^ cells were enriched from PBMC using a CD4^+^ T cell isolation kit II (Miltenyi Biotech, Bergisch Gladbach, Germany) and stained with anti-CD127 PE, CD25 APC (BD) and CD4 ECD (Beckman Coulter, Fullerton, CA, USA) antibodies. CD127^lo^CD25^+^CD4^+^ cells (CD127^lo^T_reg_) were sorted to more than 94% purity using a FACS Aria (BD) cell sorter and expanded for 14 days *in vitro* with anti-CD3/CD28 beads (Invitrogen, Invitrogen Dynal, Oslo, Norway) and 1000 U/mL recombinant human IL-2 (Chiron, Uxbridge, UK). After the expansion, the phenotype and suppressive function were assessed and cells were cryopreserved until needed.

### In vitro *cellular proliferation and apoptosis assay*

Human PMBC have been labeled with 10 μM CFSE (Invitrogen) and incubated with anti-CD3/CD28 beads (Invitrogen) in 5:1 cell per bead ratio for 5 days. 10 nM rapamycin (Sigma), *ex vivo* expanded autologous human CD127^lo^Treg (1:10 T_reg_ to PBMC ratio) or a combination of both were added to some of the cultures. After 5 days of incubation, the cells have been washed and resuspended in Annexin V binding buffer and stained with Annexin V APC, anti-CD8 APC-Cy7 (all BD), anti-CD4 ECD (Beckman Coulter) and anti-CD3 Pacific Blue (eBioscience) antibodies. Flow cytometric data were obtained using a FACS Canto II (BD) and analyzed using the FASC Diva software (BD). For accurate cell counts Calibrite beads (BD) were added to each sample.

### Human IFNG assay

We measured human IFNG in mouse serum by using a FlowCytomix fluorescent beads immunoassay according to manufacturer's instructions (Bender Medsystems GmbH, Vienna, Austria).

### Statistical analysis

All statistical analyses were performed using the nonparametric two-tailed Mann–Whitney test. The difference between the groups was considered to be statistically significant at p ≤ 0.05.

## Results

**Figure 1:**
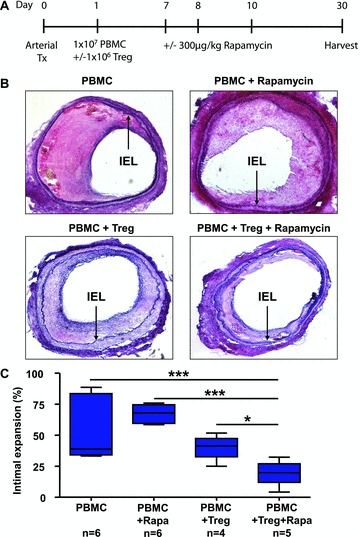
Low-dose rapamycin potentiates the inhibitory effects of T_reg_ on TA development (A) Experiment set-up: Side branches of human internal mammary artery were transplanted into immunodeficient BALB/c Rag2^−/−^Il2rg^−/−^ mice. The recipients were injected the following day with 10 × 10^6^ allogeneic human PMBCs administered i.p. Some of the mice additionally received 1 × 10^6^ *ex vivo* expanded CD127^lo^ T_reg_-injected i.p. at the same time as the PMBCs or 300 μg/kg rapamycin injected i.p. on days 7, 8 and 10 after the transplant. A fourth group of mice received a combination of PBMCs, T_reg_ and rapamycin. The arterial grafts, blood and spleen were collected 30 days after the surgery. (B) Representative photomicrographs showing development of TA in the PBMC (n = 6); PBMC and rapamycin (n = 6); PBMC and T_reg_ (n = 4); PBMC and T_reg_ and rapamycin (n = 5) groups. The grafts have been stained with Elastin/van Gieson. The elastic lamina in the media stain purple and the cellular cytoplasm pink. The newly formed neointima is delineated by the internal elastic lamina (IEL) and the vascular lumen. (C) Quantification of TA expressed as luminal occlusion, percentage of the area inside the IEL occupied by the neointima. The box plots show median, 25th and 75th percentiles as well as the highest and lowest values. *p < 0.05, ***p < 0.001.

Side branches of human IMAs, collected from patients undergoing cardiac bypass surgery, were transplanted as infrarenal interposition grafts into immunodeficient BALB/cRag2^−/−^IL2rg^−/−^ mice. The following day, recipient mice were reconstituted i.p. with 10 × 10^6^ human PBMC isolated from healthy blood donors. The PBMC donors were allogeneic to the vessel donors, as determined by HLA typing (not shown). The arterial grafts were harvested 30 days after transplantation for analysis (Figure 1A). Transplantation of a human vessel into an otherwise immunodeficient mouse reconstituted with allogeneic human PBMC results in transplant rejection as evidenced by the development of intimal expansion or TA within the graft (Figure 1B).

**Figure 2:**
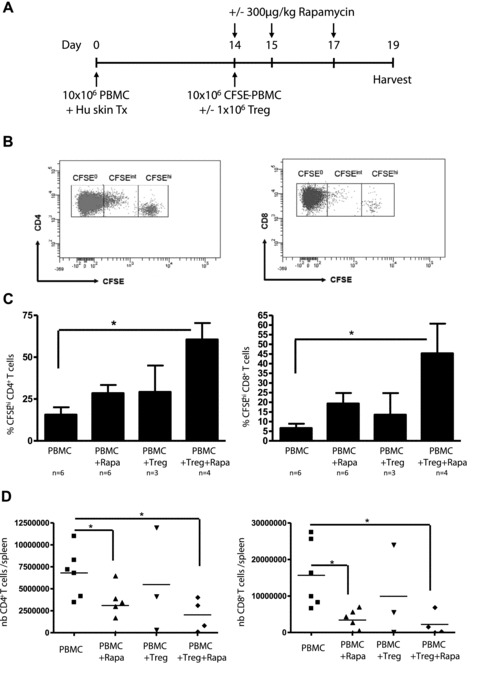
T_reg_ and rapamycin inhibit lymphocyte proliferation *in vivo* (A) Experiment setup: We injected immunodeficient BALB/c Rag2^−/−^Il2rg^−/-^ mice with 10 × 10^6^ human PMBCs i.p. and allowed 14 days for cellular reconstitution. On day 14, 10 × 10^6^ CFSE-labeled human PMBCs isolated from the same blood donor were administered i.v. alone or together with 1 × 10^6^ *ex vivo* expanded CD127^lo^ T_reg_. Some of the mice also received 300 μg/kg rapamycin i.p. on days 14, 15 and 17. Spleens were recovered on day 19 and the different cellular populations were analyzed by flow cytometry. (B) Representative flow cytometry plots demonstrating the gating strategy for CFSE high (CFSE^hi^), CFSE intermediate (CFSE^int^) and CFSE negative (CFSE^0^) CD4 and CD8 T lymphocytes in the spleen. (C) Percentage of CFSE^hi^ cells of the total CFSE^+^ CD4 and CD8 T lymphocytes in the spleen at the time of recovery in the PBMC (n = 6); PBMC and rapamycin (n = 6); PBMC and T_reg_ (n = 3); PBMC and T_reg_ and rapamycin (n = 4) groups. The error bars represent standard deviation. (D) Numbers of human CD4^+^ and CD8^+^ T lymphocytes expressed as total number of cells per spleen in the four groups. *p < 0.05.

It has previously been shown that rapamycin monotherapy at a daily dose of 500 μg/kg administered for 28 days prevented TA development in a similar mouse model of human arterial transplantation (14). For the purpose of our study, we defined a subtherapeutic dose that does not prevent the development of TA in this humanized mouse model when administered as monotherapy. Transplant recipients were either left untreated or were treated with 300 μg/kg rapamycin injected i.p. on day 7, 8 and 10 after transplantation (Figure 1A). We elected to begin rapamycin therapy at day 7 after surgery as the drug has been reported to adversely impact wound healing and to ensure that engraftment of human PBMC would not be affected during the first 7 days after transplantation. Treatment with the suboptimal dose of rapamycin did not have a significant impact on the degree of intimal expansion that developed within the transplanted human vessels in mice reconstituted with allogeneic PBMC (Figures 1B and C). However, the suboptimal dose of rapamycin did impact the ability of CD4^+^ and CD8^+^ T cells within the PBMC to respond to alloantigen *in vivo*, as demonstrated in parallel experiments where BALB/c*Rag2^−/−^IL2rg^−/−^* mice were reconstituted with 10 × 10^6^ PBMC and transplanted with allogeneic human skin. We have previously shown that in this model the CD4^+^ and CD8^+^ T cells proliferate in response to alloantigen and consistently reject the skin grafts (9). Fourteen days after skin transplantation, 10 × 10^6^ PBMC isolated from the same blood donor were labeled with CFSE and injected i.v. into the recipient mice. We chose to inject the CFSE-labeled PBMC in fully reconstituted mice to avoid the effects of homeostatic proliferation. The mice were treated with 300 μg/kg rapamycin on day 14, 15 and 17 or left untreated. Spleens were collected 2 days after the final treatment with rapamycin, on day 19, and CFSE levels in the CD4^+^ and CD8^+^ T-cell populations were analyzed by flow cytometry (Figure 2A). We quantified undivided CFSE-labeled cells as the percentage CFSE^hi^ cells of the total CFSE positive population (Figure 2B). A higher percentage of undivided CFSE^hi^ CD4^+^ and CD8 T^+^ cells (Figure 2C) and reduced total lymphocytes numbers in the spleen (Figure 2D) were observed in the rapamycin treated group, suggesting that T cells had a reduced proliferative capacity in mice treated with this rapamycin regimen compared to the untreated controls.

We have previously demonstrated that *ex vivo* expanded human CD127^lo^CD25^+^CD4^+^FOXP3^+^ T cells with regulatory activity (CD127^lo^T_reg_) can prevent the rejection of human vessel allografts (10). CD127^lo^T_reg_ almost completely abrogated TA development within the graft 30 days after transplantation when administered at a 1:1 or 1:5 CD127^lo^T_reg_:PBMC ratio (10). However, when we injected 1 × 10^6^ CD127^lo^T_reg_ together with 10 × 10^6^ PBMC from the same donor as the T_reg_ (1:10 ratio), the *ex vivo* expanded T_reg_ only had limited impact on TA (Figures 1B and C). At this ratio (1T_reg_:10PBMC), T_reg_ therapy resulted in a low level of inhibition of T-cell proliferation and IFNG production *in vivo* as demonstrated by a slower rate of proliferation of CFSE-labeled PBMC in response to alloantigen when they were injected together with CD127^lo^T_reg_ at the 10:1 ratio (Figures 2A and C). The levels of human IFNG in the plasma of arterial graft recipients were lower in mice reconstituted with a combination of PBMC and CD127^lo^T_reg_ compared to mice receiving PBMC alone (median [range] 121.5 [96–536] pg/mL vs. 283 [65–1431] pg/mL; p = 0.21) 30 days after transplantation. Of note, in our preliminary experiments the effector T cells (sorted as CD127^+^CD25^−^CD4^+^ cells) expanded in parallel to the T_reg_ cells induced significant TA when injected at 10 × 10^6^ per mice (data not shown).

To investigate the hypothesis that short-term *in vivo* therapy using a low dose of rapamycin could potentiate the functional activity of human CD127^lo^T_reg_ *in vivo*, we treated transplanted mice receiving 10 × 10^6^ PBMC and a suboptimal number of *ex vivo* expanded CD127^lo^T_reg_ from the same donor as PBMC (1:10 ratio) with 300 μg/kg rapamycin on days 7, 8 and 10 posttransplant (Figure 1A). The addition of rapamycin to the protocol significantly reduced the level of intimal expansion in the transplanted human arterial grafts (Median [CI] 19.8%[11.9–26.9] in the PBMC + T_reg_+ rapamycin group vs. 41.4%[28.7–49.5] in the PBMC + T_reg_ group; p < 0.05; Figures 1B, C). IFNG production was suppressed to almost undetectable levels in mice receiving a subtherapeutic dose of CD127^lo^T_reg_ and rapamycin (median [range] 2.3 [0.0–3.9] pg/mL; p < 0.01 vs. PBMC only and p < 0.05 vs. PBMC+ CD127^lo^T_reg_; data not shown). Moreover, the proliferation of CFSE-labeled CD4^+^ and CD8^+^ T cells in response to alloantigen *in vivo* was potently inhibited by the combination treatment compared to untreated control mice reconstituted with human PBMC alone (Figure 2B, C). Importantly, T_reg_ and rapamycin co-treatment resulted in a decrease in the total number of human lymphocytes present in the spleen (Figure 2D), suggesting its ability to modulate an already ongoing immune response.

**Figure 3:**
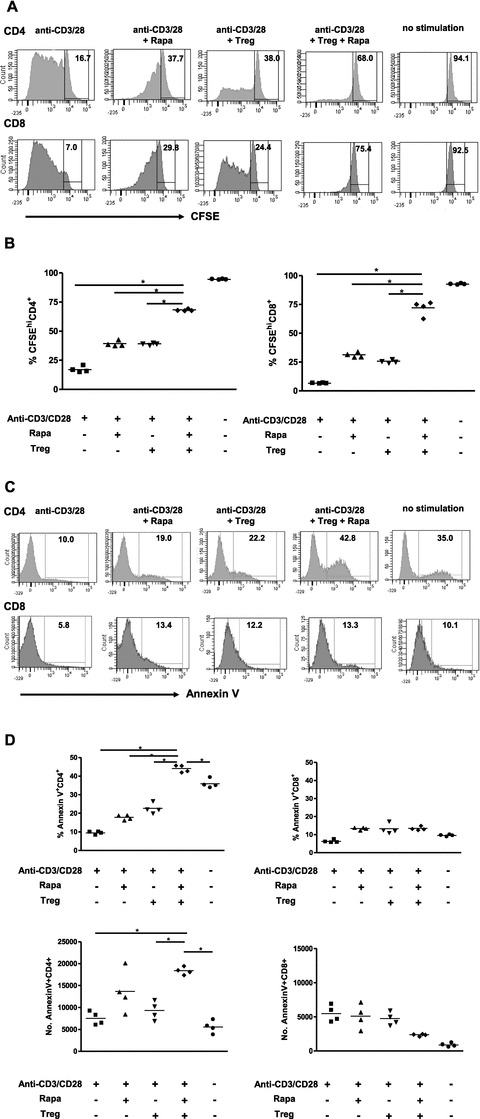
Combined therapy with T_reg_ and rapamycin inhibits CD4^+^ and CD8^+^ T-cell proliferation and potentiates apoptosis of CD4+ lymphocytes *in vitro* CFSE-labeled PBMC (10^5^ per well) have been incubated with anti-CD3/anti-CD28 beads (cells:bead ratio 5:1) in the presence or absence of 10 nM rapamycin and/or 10^4^ *ex vivo* expanded CD127^lo^ T_reg_ cells per well. (A) Representative plots depicting CFSE dilution in CD4^+^ and CD8^+^ lymphocytes after 5 day of culture. The numbers represent percentage of undivided CFSE^hi^ cells in the gated populations. (B) Percentage of undivided CFSE^hi^ lymphocytes of the total lymphocyte population, as shown in A. (C) Representative plots demonstrating the binding of the apoptosis marker Annexin V to CD4^+^ and CD8^+^ T cells. The numbers represent percentage of AnnexinV^+^ cells of the total lymphocyte population. (D) Absolute numbers and percentage of AnnexinV^+^ apoptotic cells within the CD4^+^ and CD8^+^ gates as demonstrated in panel C.

To verify the effects of T_reg_ and rapamycin on human T-lymphocyte proliferation *in vitro* we incubated CFSE-labeled human PBMC (10^5^ per well) with anti-CD3/anti-CD28 beads (cells:beads ratio 5:1) to stimulate proliferation. CD127^lo^T_reg_ were added to the cultures at a ratio of 1:10 T_reg_:PBMC with or without 10 nM rapamycin. 10 nM rapamycin corresponds to an *in vivo* concentration of 9.1 ng/mL, situated within mid-range of trough levels in rapamycin-treated transplant patients (3–18 ng/mL). Unstimulated PBMC and anti-CD3/anti-CD28 bead-stimulated PBMC receiving no additional treatment served as controls. CFSE levels were measured by flow cytometry after 5 days of culture and the degree of proliferation was expressed as the percentage of undivided CFSE^hi^ cells remaining in culture relative to the total cell population (Figures 3A, B). The presence of a suboptimal number of CD127^lo^T_reg_ and low dose of rapamycin significantly inhibited the proliferation of both CD4^+^ and CD8^+^ T cells *in vitro* compared with either treatment alone (Figures 3A, B).

The impact of combined treatment with CD127^lo^T_reg_ and rapamycin may not only result in inhibition of T-cell proliferation in response to stimulation with alloantigen, but also T-cell apoptosis. Cells undergoing apoptosis expose on their surface phosphatidylserine which is recognized by Annexin V. We measured Annexin V binding to CD4^+^ and CD8^+^ T cells stimulated with anti-CD3/anti-CD28 beads in the presence or absence of CD127^lo^T_reg_ and rapamycin (Figure 3C). Interestingly, although neither CD127^lo^T_reg_ cells nor rapamycin, either alone or in combination, had an effect on CD8^+^ T cells apoptosis, CD4^+^ cells were more susceptible to treatment-induced apoptosis. Combination treatment with CD127^lo^T_reg_ and rapamycin induced apoptosis in more than 40% of stimulated CD4^+^ lymphocytes (Figure 3D).

## Discussion

The mTOR inhibitor rapamycin promotes T_reg_ expansion both *in vitro* and *in vivo* (15–17). We have previously shown that *ex vivo* expanded CD127^lo^T_reg_ are able to prevent the development of TA in a humanized mouse model of arterial transplantation. However, the effect of the treatment was dose-dependent and efficiency was lost when using suboptimal numbers of T_reg_ (10). Here, we hypothesized that short treatment with low-dose rapamycin would promote the ability of T_reg_ to inhibit TA. We demonstrate that T_reg_ and rapamycin inhibit the proliferation of CD4^+^ and CD8^+^ T cells both *in vivo* and *in vitro* and induce apoptosis of CD4^+^ T cells *in vitro*. Combination treatment of transplant recipients with low-dose rapamycin and subtherapeutic numbers of CD127^lo^T_reg_ led to significantly reduced intimal expansion in the arterial allografts compared to subtherapeutic T_reg_ treatment alone. The inflammatory cytokine IFNG, which plays an important role in the pathogenesis of TA, was inhibited to almost undetectable levels in the serum of mice receiving the combination of T_reg_ and rapamycin.

Due to their natural immunosuppressive abilities, T_reg_ have emerged in recent years as a viable alternative to control immune responsiveness to transplanted allogeneic cells and tissues (18). The immunosuppressive regimens currently used in clinical transplantation lack immunological specificity and are therefore associated with serious side effects such as infections and increased risk for malignancy. T_reg_s have the advantage of being a physiological cell population with the capacity to respond to alloantigen (19). Several protocols designed for T_reg_ cell therapy in cell and organ transplantation and other immune-mediated disorders are currently being investigated in laboratory studies and clinical trials (7). However, despite improved isolation and expansion protocols, generation of large numbers of T_reg_ needed to prevent rejection of solid organ allografts remains an ongoing challenge (8).

An attractive therapeutic alternative would be to associate T_reg_-based cellular immunotherapy with other biological or chemical immunosuppressants. However, most of the immunosuppressive regimens currently used in clinical practice, particularly those utilizing calcineurin inhibitors, have the potential to inhibit T_reg_ survival and proliferation alongside that of conventional T cells that mediate rejection (20). In contrast, rapamycin has been demonstrated to inhibit effector T-cell proliferation but not to interfere with the STAT5 pathway, which is preferentially used by T_reg_s (5, 21). In addition, recent data demonstrate that transient mTOR inhibition with rapamycin followed by TCR stimulation promotes preferential T_reg_ proliferation compared to conventional T cells (12,13). Moreover, the number of CD4^+^CD25^+^FOXP3^+^ T cells was found to be significantly higher in transplant patients treated with rapamycin in comparison to recipients receiving cyclosporine (16,17,22). Considering all of the above, rapamycin may be the drug of choice for use in combination with T_reg_ therapy in transplantation.

In our study, we have demonstrated the combined inhibitory effect of T_reg_ and rapamycin on CD4^+^ and CD8^+^ T-cell proliferation *in vivo* and *in vitro*. Unexpectedly, in our *in vitro* experiments only CD4^+^ T-cell apoptosis was accelerated by the treatment. This may be due to a differential expression of Pim kinases which provide an mTOR independent pathway to promote survival of proliferating cells (23) and have been demonstrated to promote survival of T_reg_ (24) and primed CD8^+^ T cells upon CD27 costimulation (25).

Rapamycin seems to be particularly effective to inhibit TA both in experimental and clinical studies. The development of TA is the main pathologic feature associated with chronic transplant dysfunction, as the neointima gradually obstructs the vascular lumen leading to organ ischemia (26). In long-term survivors of heart transplantation, arteriosclerosis of the coronary vasculature is the second most common cause of death after malignancy (27, 28). Immunosuppressive therapy with antiproliferative agents (azathioprine and mycophenolate) or calcineurin inhibitors (cyclosporine and tacrolimus) has limited efficiency in preventing chronic transplant dysfunction due to TA (29). In experimental studies, high-dose rapamycin monotherapy was previously shown to inhibit IFNG production and TA development in a humanized mouse model (14). In addition, rapamycin has direct inhibitory effects on smooth muscle cells proliferation and migration, which are important components of neointima formation in TA (30,31). Clinically, rapamycin was shown to improve coronary flow reserve and microcirculatory resistance measured at 1 year after transplantation (32). In patients with advanced coronary allograft vasculopathy conversion to rapamycin significantly slowed disease progression compared to other imunosuppressive regiments (33,34). Other authors report the ability of rapamycin to induce regression of already present advanced coronary lesions in a 33-year-old heart transplant recipient (35). Comparative studies have indicated superior efficacy and reduced side effects of rapamycin over the previously used immunosuppressive therapies in heart transplant recipients (34).

The humanized mouse model of arterial transplantation, based on rejection of human arterial grafts by allogeneic human PBMC in immunodeficient hosts, provides an experimental setting which is closer to the clinical scenario compared to mouse-to-mouse transplantation. However, this model is unlikely to accurately reflect the complex immune interactions that occur in human transplant recipients. The results presented here need therefore to be interpreted with due caution and cannot be directly extrapolated to the clinical setting. It should be stressed that the rapamycin dose used in our *in vivo* experiments (300 μg/kg) which was demonstrated to be subtherapeutic both in a humanized model and in a mouse model of aortic transplantation (Nadig SN, unpublished) is much higher than the therapeutic dose used in clinical transplantation (the commonly used 6 mg loading dose of rapamycin equals 75 μg/kg for an 80 kg person). However, direct comparison of dosing may not be appropriate as it has been reported that a single 8 mg/kg dose of rapamycin given to mice resulted in a 12–40 ng/mL serum level (36), therefore, more than 100 times higher doses are required in mice to achieve serum levels similar to rapamycin trough levels observed in transplant patients (3–18 ng/mL). However, the beneficial effects of rapamycin-based immunosuppressive regimens on coronary allograft vasculopathy in heart transplant patients suggest that mechanisms similar to those described in our study might occur in human solid organ recipients. Further studies are required to determine whether the beneficial effects of the drug are due to the observed increase in T_reg_ numbers in rapamycin-treated patients or to local antiproliferative and antimigratory effects on smooth muscle cells in the arterial wall.

Here, we provide proof of concept that short treatment using low-dose rapamycin can potentiate the previously demonstrated ability of T_reg_ to inhibit TA development in a chimeric humanized mouse system of arterial transplantation. TA reduction was associated with a combined suppressive effect of T_reg_ and rapamycin on effector T-cell proliferation and IFNG production. These data have potential implications for clinical practice as rapamycin is already an approved drug for clinical use and T_reg_ therapy is currently being tested in clinical trials. The combination treatment would allow clinicians to boost the efficacy of T_reg_-based treatment protocols and at the same time to lower the currently used doses of rapamycin, thus minimizing its side effects. By inhibiting arterial neointima formation, T_reg_ and rapamycin may prevent chronic transplant dysfunction, leading to improved long-term allograft survival.

## Abbreviations

CFSE: carboxyfluorescein diacetate succinimidyl ester
IFNG: interferon gamma
IMA: internal mammary artery
i.p.: intraperitoneally
i.v.: intravenously
mTOR: mammalian target of rapamycin
PBMC: peripheral blood mononuclear cell
TA: transplant arteriosclerosis
T_reg_: regulatory T cells
